# Hospital safety culture in Australia: a nationwide survey using a safety attitude questionnaire

**DOI:** 10.1111/avj.13474

**Published:** 2025-07-10

**Authors:** LCP Santos, N Perkins, W Goodwin

**Affiliations:** ^1^ School of Veterinary Sciences The University of Queensland Brisbane Queensland Australia; ^2^ Present address: School of Biodiversity, One Health & Veterinary Medicine The University of Glasgow Bearsden UK

**Keywords:** Australia, patient safety, safety attitudes questionnaire, safety culture, veterinary medicine

## Abstract

**Background:**

Patient safety culture is increasingly recognised as important in veterinary medicine; however, there is limited understanding of how safety attitudes vary across professional roles within Australian veterinary practices. This study investigates the perceptions of safety culture, focusing on its importance for enhancing workplace well‐being and patient safety.

**Methods:**

A cross‐sectional survey was conducted among 669 Australian veterinary care professionals across diverse practice types, roles and locations. The Safety Attitudes Questionnaire (SAQ) assessed six dimensions: teamwork climate, safety climate, job satisfaction, stress recognition, perceptions of management and working conditions. Responses were collected on a Likert scale and analysed to compare perceptions across professional roles.

**Results:**

Overall positive attitudes were highest for Stress Recognition (63.4%) and lowest for Working Conditions (25.4%). Managers reported significantly more positive attitudes than veterinarians and nurses across multiple dimensions, including teamwork climate (χ^2^ = 29.1, P < 0.001) and perceptions of management (χ^2^ = 31.1, P < 0.001). Academic clinicians reported notably low attitudes, with only 13.3% scoring positively for safety climate and none for perceptions of management. Comparisons between veterinarians and nurses revealed significant differences in stress recognition (Z = −6.0, P < 0.001), perceptions of management (Z = −2.1, P = 0.04) and working conditions (Z = −2.4, P = 0.01), with veterinarians consistently reporting higher scores.

**Conclusions:**

Significant variability exists in safety attitudes across professional roles, with managers reporting the most favourable perceptions and academic clinicians and nurses reporting the least. Veterinarians also scored higher than veterinary nurses for several dimensions.

Patient safety has become a focal point in human healthcare research, gaining prominence following the Institute of Medicine's seminal report,^1^ which highlighted the prevalence of medical errors and their impact on patient outcomes. In this context, safety culture – defined as the shared values, beliefs and norms about the importance of safety within an organisation – has emerged as a critical factor in reducing adverse events.^2^ Patient safety, in contrast, is the outcome of these cultural attributes – reflected in the prevention of harm to patients during the delivery of care.^3^ In this context, a strong safety culture is foundational to achieving high standards of patient safety. Key elements of a strong safety culture include effective teamwork, open and effective communication and a commitment to continuous learning and improvement. Robust evidence from human health care, demonstrates that fostering a positive safety culture can significantly enhance patient outcomes.^4^, ^5^, ^6^ However, in veterinary medicine, patient safety remains a relatively new and underexplored area, with limited research examining how these principles apply in veterinary settings.

The term “safety culture” first appeared after the Chernobyl nuclear power disaster in 1988,^7^ highlighting the critical role that organisational culture plays in preventing catastrophic failures. The need to cultivate a safety culture in veterinary practice has been emphasised, highlighting the profession's lag behind other safety‐critical industries, such as aviation, rail and health care.^8^, ^9^ Research has also explored the assessment of safety culture in veterinary settings, identifying gaps and opportunities for improvement.^10^, ^11^ Additionally, the development of veterinary safety culture has underscored the importance of addressing human factors and nontechnical skills to improve outcomes.^9^ Building on this foundation, the integration of human factors and systems‐thinking principles into veterinary education can offer a practical and comprehensive framework to advance patient safety.^12^

Veterinary care professionals operate across diverse settings, including small animal, large animal and mixed animal practices and referral hospitals. Each of these environments presents unique challenges that may influence perceptions of teamwork, stress recognition and safety climate. Understanding these perceptions is essential, as they directly impact patient care quality, team dynamics and workplace well‐being. This study aims to investigate the perceptions of Australian veterinary professionals regarding safety culture and to identify the factors that influence it. Specifically, we hypothesised that positive attitudes towards safety culture dimensions would vary significantly across professional roles, with managers reporting higher scores compared with other groups. This expectation was based on previous research suggesting that individuals in managerial positions often have greater involvement in safety initiatives and a broader perspective on organisational processes, which may lead to more favourable perceptions of safety culture.^13^, ^14^ We anticipated that managers would report higher scores particularly in the dimensions of teamwork climate, safety climate and perceptions of management. As a result, they may perceive team dynamics, safety practices and leadership effectiveness more positively than frontline staff, who may experience these aspects differently in day‐to‐day practice. Additionally, we hypothesised that veterinarians and veterinary nurses would differ significantly in their attitudes towards all safety culture dimensions. Veterinarians typically hold decision‐making authority and are responsible for diagnosis, treatment planning and client communication, which may shape their perceptions of safety culture differently from veterinary nurses, who often focus on patient care, monitoring and supporting clinical procedures. These role‐based differences can influence how each group perceives and prioritises safety practices. Ultimately, this study contributes to the growing body of knowledge on veterinary patient safety, providing insights into the complexities of safety culture in veterinary practice and establishing a foundation for future research in this field.

## Materials and methods

### Study design

This study was conducted following ethical approval from The University of Queensland Human Research Ethics Committee, Approval Number: 2021/HE000324. Details of this internet‐based survey are reported in accordance with the CHERRIES guidelines to ensure clarity, reproducibility and integrity. The study employed a cross‐sectional, web‐based survey design targeting professional roles in Australian veterinary practices. Incomplete or duplicate responses were excluded. The survey was closed and accessible only to eligible participants who received an invitation, ensuring that only the intended population could participate. Measures were implemented to prevent multiple submissions from the same individual, such as disabling the ability to retake the survey from the same device.

Participation in this survey was voluntary, and no incentives were offered to respondents. The survey was designed to respect participants' privacy and confidentiality by automatically anonymising responses. After drafting the survey instrument, a pilot test was conducted with a small group of veterinary professionals representing a diverse range of roles, including veterinarians, nurses and practice managers. Feedback from the pilot participants focused on the clarity of survey items, ease of completion and overall length of the survey. Minor modifications were made to address ambiguities and improve the user experience. For example, some items were revised to reflect terminology more commonly used in the veterinary setting. The pilot also confirmed the appropriateness of the estimated completion time and the feasibility of the online format for large‐scale deployment.

### Sample population and recruitment

We employed a convenience sampling method to recruit participants from diverse roles and experience levels within Australian veterinary practices. Recruitment efforts included distributing email invitations to 2495 veterinary practices; making announcements through professional associations representing veterinarians, veterinary nurses and practice managers; and using targeted social media advertisements. This multichannel approach aimed to ensure broad representation across staff roles, grades and levels of experience. Although invitations were sent to practices, responses were collected from individual veterinary professionals. The survey design allowed for multiple individuals from the same practice to participate, and a response rate is unknown. The inclusion criteria required participants to be currently employed in veterinary practices in Australia. Sample size estimates were calculated using an online tool for a proportion with specified precision (https://epitools.ausvet.com.au/oneproportion). Estimates assumed an estimated true proportion of 5% and 10%, 95% confidence level, a desired precision of 5% or 10% and a population size for finite populations ranging from 50 to 500 and 1000 for the different strata. The survey was administered via the Qualtrics online survey platform, which allowed for secure data collection and management. The survey was accessible online from November 2021 to February 2022.

### Survey instrument

**Figure 1:**
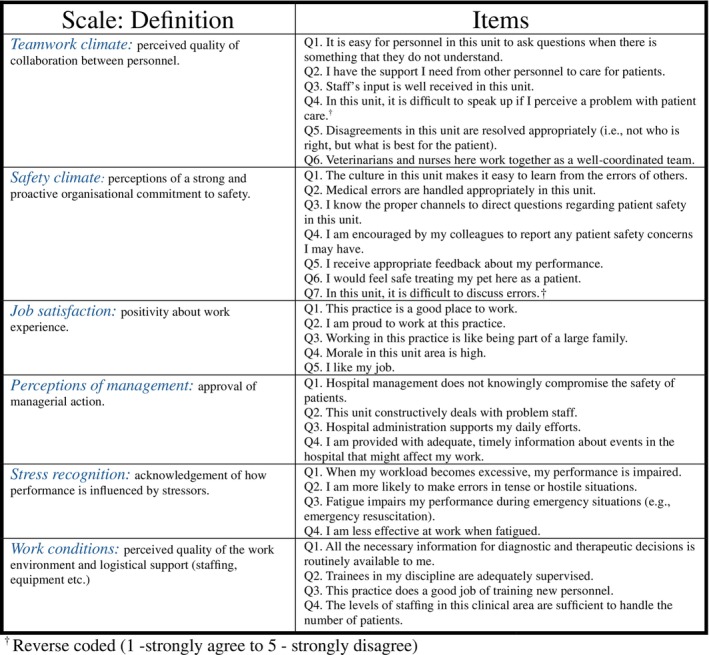
Safety Attitudes Questionnaire (SAQ) factor definitions and items, as administered to Australian veterinary professionals (n = 669).

The survey questionnaire comprised a total of 36 items, including demographic questions and standardised scales measuring safety culture. Safety culture was assessed using a validated tool (Safety Attitudes Questionnaire, SAQ) developed by Sexton et al.^15^ The SAQ evaluates six key dimensions relevant to safety culture: teamwork climate, safety climate, job satisfaction, stress recognition, perception of management and working conditions. At the time of study design and ethics approval, the veterinary‐adapted version of the SAQ (as later described by Love et al., 2021) had not yet been published. Therefore, we used the original tool, which is widely validated in human health care. This choice enabled direct comparison with human healthcare data and contributed to the validation of the SAQ's applicability in veterinary contexts. Example items can be seen in Figure 1. Responses were recorded on a 5‐point Likert scale ranging from 1 (strongly disagree) to 5 (strongly agree), with some items requiring reversed scoring.

The scoring system used in our study followed the methodology established in previous research.^16^, ^17^ First, each dimension item was converted from a 5‐point Likert scale to a 100‐point scale (1 = 0, 2 = 25, 3 = 50, 4 = 75 and 5 = 100). To accomplish this, the Likert scale item values were subtracted by 1, and the resulting value was then multiplied by 25 to derive each item's scale score. The mean scores for each dimension were subsequently calculated. Finally, respondents with a mean score of 75 or higher were classified as holding a positive attitude towards that dimension. To further analyse the proportion of positive attitudes, binary variables were created for each dimension, where scores ≥75 were coded as 1 (positive attitudes) and scores <75 were coded as 0 (nonpositive attitudes).

### Psychometric analysis

The internal consistency reliabilities of the SAQ dimensions were assessed using Cronbach's alpha. Confirmatory factor analysis (CFA) using the two‐step structural equation model (RStudio‐Version 2024.12.0 + 467, Posit Software, PBC) was applied to test the extent to which each SAQ dimension was explained by items and the extent to which safety culture was explained by the five dimensions. A set of goodness‐of‐fit indices for the dimension structure model was used, including the comparative fit index (CFI), Tucker‐Lewis index (TLI), root mean squared error of approximation (RMSEA) and standardised root mean square residual (SRMR). Statistical significance was set at P < 0.05.

### Data analysis

Data analysis was conducted using the Statistical Package for the Social Sciences (IBM SPSS Statistics Version 28.0.0.0). The Kolmogorov–Smirnov test was used to assess the normality of the data, and all variables were found to deviate from a normal distribution, necessitating the use of nonparametric tests. Descriptive statistics were used to summarise the demographic characteristics of the respondents, including the number and frequency of responses for categorical variables. Categorical data are presented as frequency and percentage and compared using χ^2^‐test. To investigate differences in positive attitudes between professional roles, we conducted pairwise comparisons among the five professional roles using chi‐square tests of independence. In order to maintain clarity, only statistically significant pairwise results (P < 0.005) are presented. This approach allowed us to examine differences in the percentage of respondents reporting positive attitudes for each dimension. Bonferroni correction was applied to account for multiple comparisons among the five professional roles, with statistical significance set at P < 0.005.

Additionally, a Mann–Whitney *U* test was conducted to compare scores across the six safety attitudes dimensions between veterinarians and veterinary nurses, as these groups constituted most respondents [548 of 669 (82%)]. The Mann–Whitney *U* test was chosen due to the nonparametric nature of the data. For each comparison, the U‐statistic was converted to a standardised *Z*‐score, which is reported in the results (Table 5) along with the corresponding P‐values. Chi‐square tests were also used to compare responses for individual items between these two groups of professionals, focusing on the percentage of respondents reporting positive attitudes.

## Results

### Demographics of study participants

In total, 669 individual responses were obtained from staff working in 2495 invited veterinary practices across Australia. As shown in Table 1, most participants were female (571 of 669 [85%]). Most respondents worked in small animal general practices (345 of 669 [52%]), followed by mixed animal practices (141 of 669 [21%]) and small animal referral hospitals (97 of 669 [15%]). Professional roles were predominantly veterinarians (347 of 669 [52%]) and veterinary nurses (201 of 669 [30%]).

**Table 1:** Demographic characteristics of Australian veterinary professional survey respondents by type of practice, professional role, gender and location (n = 669)

Category	Subcategory	N (%)
Practice	Small animal	345 (52)
	Mixed animal	141 (21)
	Small animal referral	97 (15)
	Veterinary teaching hospital	55 (8)
	Equine referral	31 (5)
Role	Veterinarian	347 (52)
	Veterinary nurse/technician	201 (30)
	Management/director	86 (13)
	Resident/intern	20 (3)
	Academic clinician	15 (2)
Gender	Female	571 (85)
	Male	90 (14)
	Prefer not to say	8 (1)
Location	South Australia	210 (31)
	Queensland	132 (20)
	Victoria	127 (19)
	New South Wales	84 (13)
	Western Australia	82 (12)
	Tasmania	18 (3)
	Australia capital territory	9 (1)
	Northern Territory	7 (1)

### Psychometric properties of the questionnaire

The Cronbach's alpha values for the six SAQ dimensions ranged from 0.77 (working conditions) to 0.92 (job satisfaction), indicating good internal consistency across scales. CFA demonstrated strong model fit, with TLI and CFI values exceeding 0.90 and RMSEA and SRMR values below 0.10 (Table 2). Factor loadings for individual items were generally high (>0.70), supporting the validity of the dimensions and overall construct (Table 2).

**Table 2:** Psychometric properties of the Safety Attitudes Questionnaire (SAQ) administered to Australian veterinary professionals: confirmatory factor analysis and Cronbach's alpha for each dimension

SAQ dimensions	CFI	TLI	RMSEA	SRMR	Cronbach's α
Teamwork climate	0.97	0.95	0.09	0.05	0.87
Safety climate	0.98	0.97	0.07	0.02	0.89
Job satisfaction	0.98	0.95	0.14	0.03	0.92
Stress recognition	0.98	0.94	0.13	0.03	0.84
Work conditions	0.99	0.96	0.09	0.03	0.81
Perceptions of management	0.98	0.95	0.11	0.03	0.77
Overall model	0.93	0.92	0.06	0.05	

CFI, comparative fit index; RMSEA, root mean square error of approximation; SRMR, standardised root mean square residual; TLI, Tucker‐Lewis index.

### Questionnaire overall positive responsiveness per scale

Stress recognition showed the highest percentage of overall positive attitudes (424 of 669 [63.4%]), followed by teamwork climate (327 of 669 [49.0%]) and job satisfaction (327 of 669 [48.9%]). Perceptions of management (237 of 669 [35.4%]) and working conditions (170 of 669 [25.4%]) demonstrated the lowest positive attitude scores (Table 3).

**Table 3:** Australian veterinary professionals' (n = 669) perception of safety culture across Safety Attitudes Questionnaire (SAQ) dimensions: minimum, maximum, median (IQR) and percentage of overall positive attitudes

	Minimum	Maximum	Median (IQR)	% overall positive attitudes
SAQ dimensions				
Teamwork	4.2	100	70.8 (58.3–83.3)	49.0%
Safety climate	14.3	100	71.4 (53.6–78.6)	41.4%
Job satisfaction	0.00	100	70.0 (50.0–85.0)	48.9%
Stress recognition	12.5	100	75.0 (68.8–93.8)	63.4%
Perceptions of management	0.00	100	68.3 (50.0–75.0)	35.4%
Working conditions	0.00	100	56.3 (37.5–68.8)	25.4%

Scores for each dimension are presented on a scale from 0 to 100. Positive attitude defined by a mean score ≥75.

### Pairwise comparisons of positive safety attitudes across roles

**Figure 2:**
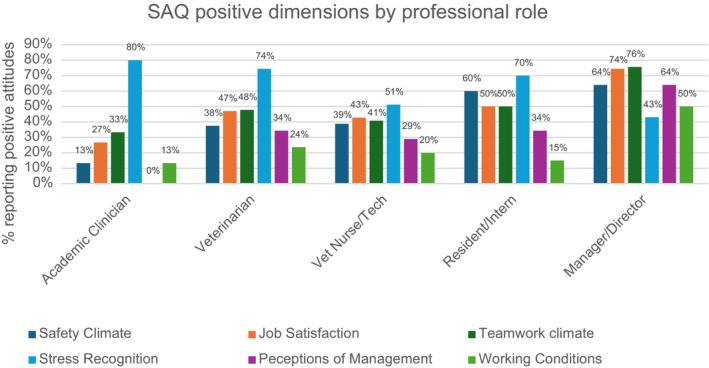
Variation in the proportion of Australian veterinary professionals (n = 669) reporting positive attitudes across six Safety Attitudes Questionnaire (SAQ) dimensions, categorised by professional role (managers/directors, veterinarians, veterinary nurses/technicians, academic clinicians and residents/interns).

Table 4 summarises only the significant results from pairwise chi‐square tests across the five professional roles (with nonsignificant comparisons omitted), Figure 2 presents the percentages of positive attitudes per role, and a separate, detailed comparison focuses on veterinarians and veterinary nurses given their larger sample sizes. Managers consistently reported significantly more positive attitudes across several dimensions when compared with other professional roles. Specifically, managers demonstrated higher scores than nurses in teamwork climate (χ^2^ = 29.1, P < 0.001), safety climate (χ^2^ = 15.3, P < 0.001), job satisfaction (χ^2^ = 24.2, P < 0.001), perceptions of management (χ^2^ = 31.1, P < 0.001) and working conditions (χ^2^ = 26.5, P < 0.001). Similarly, managers reported more positive attitudes than veterinarians for teamwork climate (χ^2^ = 21.3, P < 0.001), safety climate (χ^2^ = 19.8, P < 0.001), job satisfaction (χ^2^ = 20.8, P < 0.001), perceptions of management (χ^2^ = 25.2, P < 0.001) and working conditions (χ^2^ = 23.3, P < 0.001). Comparisons between managers and academic clinicians revealed significant differences in teamwork climate (χ^2^ = 10.7, P = 0.001), safety climate (χ^2^ = 13.3, P < 0.001), job satisfaction (χ^2^ = 13.2, P < 0.001) and perceptions of management (χ^2^ = 21.1, P < 0.001). In addition, academic clinicians scored significantly lower than residents/interns for safety climate (χ^2^ = 7.8, P = 0.005). Stress recognition scores were notably higher for veterinarians compared with nurses (χ^2^ = 30.2, P < 0.001), whereas managers also outperformed veterinarians on this dimension (χ^2^ = 31.1, P < 0.001). No significant differences were observed between academic clinicians and nurses or between residents/interns and nurses across most dimensions.

**Table 4:** Pairwise comparisons of Safety Attitudes Questionnaire (SAQ) dimensions across five veterinary professional roles (n = 669)

Dimension	Pairwise comparison	χ^2^	P‐value
Teamwork climate	Managers versus nurses	29.1	<0.001
	Managers versus veterinarians	21.3	<0.001
	Managers versus academic clinicians	10.7	0.001
Safety climate	Managers versus nurses	15.3	<0.001
	Managers versus veterinarians	19.8	<0.001
	Managers versus academic clinicians	13.3	<0.001
	Academic clinicians versus resident/interns	7.8	0.005
Job satisfaction	Managers versus nurses	24.2	<0.001
	Managers versus veterinarians	20.8	<0.001
	Managers versus academic clinicians	13.2	<0.001
Stress recognition	Veterinarians versus nurses	30.2	<0.001
	Managers versus veterinarians	31.1	<0.001
Perceptions of management	Managers versus nurses	31.1	<0.001
	Managers versus resident/interns	10.0	0.002
	Managers versus veterinarians	25.2	<0.001
	Managers versus academic clinicians	21.1	<0.001
Working conditions	Managers versus nurses	26.5	<0.001
	Managers versusveterinarians	23.3	<0.001
	Managers versusresident/interns	8.1	0.004

The χ^2^ and P‐values indicate significant differences in perceptions between roles for each SAQ dimension. Pairwise comparisons were adjusted using the Bonferroni correction (P < 0.005).

Table 5 summarises the median scores and interquartile ranges for veterinarians and veterinary nurses, specifically addressing one of our hypotheses regarding differences between these professional roles. Significant differences were identified for stress recognition (Z = −6.0, P < 0.001), perceptions of management (Z = −2.1, P = 0.04) and working conditions (Z = −2.4, P = 0.01), with veterinarians consistently reporting higher scores. However, no significant differences were observed for teamwork climate (Z = −1.8, P = 0.18), safety climate (Z = −0.3, P = 0.75) or job satisfaction (Z = −0.6, P = 0.53).

**Table 5:** Comparison of safety attitude questionnaire (SAQ) dimension scores between Veterinarians (n = 347) and Veterinary Nurses (n = 201): Median (IQR), Z Test and P‐values

Dimension	Veterinarians median (IQR)	Nurses median (IQR)	Z test	P value
Teamwork climate	70.8 (58.3–80.9)	66.7 (50.0–83.3)	−1.8	0.18
Safety climate	67.9 (53.6–78.6)	67.9 (50.0–78.6)	−0.3	0.75
Job satisfaction	70.0 (55.0–85.0)	70.0 (50.0–85.0)	−0.6	0.53
Stress recognition	81.3 (68.8–93.8)	75.0 (59.4–81.3)	−6.0	<0.001*
Perceptions of management	62.5 (50.0–75.0)	62.5 (43.8–75.0)	−2.1	0.04*
Working conditions	56.3 (43.8–68.8)	56.3 (34.4–68.8)	−2.4	0.01*

* Statistically significant when P < 0.05.

## Discussion

This study aimed to assess the safety attitudes of veterinary professionals, particularly since no national‐level data had been previously reported in Australian veterinary practice. Our results indicate that although some dimensions of safety culture were perceived positively, notable disparities emerged across professional roles – particularly concerning perceptions of management and working conditions. Stress recognition had the highest proportion of positive responses (63.4%), potentially reflecting greater awareness of psychological challenges in the veterinary profession (e.g., such as compassion fatigue and burnout).^18^ This is particularly relevant given the timing of the survey, which was conducted during the COVID‐19 pandemic. Previous research has shown that the pandemic significantly increased stress and psychological strain among veterinary professionals. For example, McKee et al.^19^ reported heightened psychosocial demands and reduced well‐being among veterinary academics, whereas Quain et al.^20^ found that veterinary teams faced more ethically challenging situations and moral stress due to pandemic‐related constraints. These findings support the interpretation that the pandemic context may have influenced participants' responses, particularly in relation to stress and workplace well‐being. Teamwork climate (49%) and job satisfaction (48.9%) followed, suggesting moderate levels of interprofessional collaboration and workplace satisfaction. In contrast, perceptions of management (35.4%) and working conditions (25.4%) were the least favourable, indicating that some frontline staff perceive a lack of managerial support and inadequate resources for daily operations. These findings highlight the need for stronger managerial engagement and clearer communication to address staff concerns regarding organisational support and resource allocation.^21^ Veterinary medicine has been slower to adopt a structured approach to patient safety culture compared with other safety‐critical industries (e.g., human health care, aviation, nuclear power).^8^, ^22^ Without a system‐wide commitment to patient safety, efforts to enhance safety culture may remain fragmented, highlighting the need for leadership engagement and resource allocation to address these challenges.^8^

A key objective was to examine whether attitudes towards safety culture varied by professional role. Managers consistently reported more positive perceptions across multiple dimensions, including teamwork climate, safety climate and perceptions of management. This trend aligns with previous findings in human health care, where managerial staff often exhibit more optimistic views due to their involvement in strategic decision‐making and reduced exposure to frontline operational challenges.^4^, ^23^, ^24^ Singer et al.^25^ identified significant discrepancies in safety climate perceptions, with nurses reporting less favourable views of senior management engagement and resource availability compared with physicians. Leadership engagement plays a central role in fostering a positive safety culture, particularly by promoting psychological safety and encouraging the reporting of safety concerns.^8^ Failure of veterinary leaders to actively involve frontline staff in decision‐making may create a disconnect between managerial perspectives and the lived experiences of clinical staff, which could explain the lower ratings of perceptions of management in this study. These findings suggest that improving transparency in leadership decisions, ensuring equitable resource distribution and enhancing communication between management and clinical staff could strengthen the overall safety climate. Notably, none of the academic clinicians in our sample reported a positive attitude towards the “perceptions of management” dimension. Although this result should be interpreted with caution due to the small sample size for this group, it may reflect unique challenges within academic institutions, such as more complex organisational hierarchies, differing expectations between clinical and academic leadership, or perceived disconnects between frontline staff and institutional management. This finding highlights the need for further research focused specifically on safety culture and management perceptions within academic veterinary environments.

Another key objective was to explore differences in perceptions between veterinarians and veterinary nurses. Veterinarians reported higher scores in stress recognition, perceptions of management and working conditions. These differences may be attributed to veterinarians' greater autonomy in clinical decision‐making and advanced professional training, equipping them with enhanced coping strategies for workplace stressors. Conversely, veterinary nurses, who are often at the “sharp end” of patient care, may experience higher levels of workplace strain due to their direct engagement in patient handling, procedural support and administrative duties. This finding aligns with studies highlighting increased burnout risk, workload pressure and limited decision‐making authority among veterinary nurses and technicians, particularly in teaching hospitals where staffing ratios are often inadequate and emotional labour is high.^26^, ^27^, ^28^, ^29^ Furthermore, research has shown that perceptions of management and teamwork climate vary significantly by professional role. For instance, a study in a US veterinary teaching hospital found that veterinary technicians voiced greater concerns regarding staffing shortages, workload demands and communication breakdowns compared with faculty/house officers (veterinarians), suggesting possible disparities in managerial support.^11^ These findings align with human healthcare literature, where nurses frequently report lower safety climate scores than physicians.^30^ This discrepancy has been linked to increased exposure to operational challenges, higher workload demands and limited decision‐making authority, which may also apply to veterinary nurses. Despite these differences, teamwork climate, safety climate and job satisfaction did not significantly differ between veterinarians and veterinary nurses in our study, suggesting that collaborative workplace dynamics play a key role in shaping safety culture across professional roles. Although perceptions of workload and management may diverge, both groups consistently endorsed teamwork and mutual support as important features of workplace safety.^11^ Creating a positive safety culture requires engagement from all levels of an organisation and without inclusive leadership and systemic improvements, professional role disparities in safety perceptions may persist.^5^ In Love et al.^11^ both veterinary technicians and veterinarians highlighted teamwork and mutual support as key to patient safety, suggesting that workplace culture can transcend formal job titles within at least one US teaching hospital setting.

These findings underscore the need for targeted interventions to address identified gaps among veterinary professionals. Given their more favourable perceptions, managers are well positioned to lead safety initiatives, but their engagement with frontline perspectives should be strengthened to bridge existing perception gaps. Our study suggested that academic clinicians may benefit from organisational support that facilitates a better balance between teaching, research and clinical responsibilities while integrating them into safety‐related decision‐making processes. Similarly, veterinary nurses would likely benefit from structured career progression pathways, formal recognition of their contributions and strategies aimed at alleviating workload‐related stress.

A major strength of this study is its adaptation of a validated human healthcare tool for veterinary practice, allowing for meaningful cross‐disciplinary comparisons in safety climate research. Our findings further show that the SAQ, used by Australian veterinary professionals, possesses strong psychometric properties. Specifically, Cronbach's alpha values between 0.77 and 0.92 align with the reliability seen in human health care.^15^ In addition, confirmatory factor analysis further reinforces the structural validity of the instrument, as evidenced by acceptable thresholds in TLI, CFI, RMSEA and SRMR. Together, these results highlight the SAQ's value as a robust measure of safety culture within veterinary settings.

However, several limitations should be acknowledged. First, the sample was limited to Australian veterinary professionals, which may constrain the generalisability of the findings to other regions with different workplace cultures, leadership structures or regulatory environments. Furthermore, this study was conducted between November 2021 and February 2022, during a period when the COVID‐19 pandemic was still affecting veterinary workplaces in Australia. Although we did not specifically analyse the impact of the pandemic on safety culture perceptions, it is possible that heightened stress levels, changes in team dynamics and altered communication practices during this time may have influenced participant responses—particularly in dimensions such as stress recognition and teamwork climate. This should be considered when interpreting the findings.

Second, the study employed a cross‐sectional design, providing a snapshot of safety attitudes at a single point in time. This limitation restricts the ability to draw causal inferences regarding the impact of specific factors on safety culture perceptions. Future research employing longitudinal designs could offer deeper insights into how safety attitudes evolve over time and how targeted interventions influence key dimensions such as perceptions of management and working conditions. Additionally, qualitative investigations could complement quantitative findings by exploring the underlying reasons for role‐based differences in safety attitudes and identifying context‐specific barriers to safety culture improvement.

Third, although 2495 veterinary practices were contacted and 669 individual responses were received, the actual number of unique practices represented is unknown, and the reported response rate reflects individual‐level participation rather than practice‐level engagement. It is possible that individuals with particularly strong views—positive or negative—were more likely to complete the survey, which may have influenced overall trends.

Fourth, the survey did not capture whether respondents who identified as managers also held clinical roles, such as veterinarians or veterinary nurses. This overlap may have influenced how these individuals responded to questions related to safety culture. Future studies should consider collecting more detailed role information to better account for dual responsibilities in data interpretation.

Finally, several subgroups in our sample had small numbers of respondents, including residents and interns (n = 20), academic clinicians (n = 15) and managers (n = 86). These small subgroup sizes reduce the statistical power of comparative analysis and limit the generalisability of findings related to these specific roles. Future research could seek to improve representation from smaller professional subgroups and use mixed methods to better understand barriers to participation.

In conclusion, this study confirms the applicability of the SAQ for measuring safety culture in veterinary settings. Our findings show that professional roles significantly shape safety perceptions, with managers generally reporting more favourable attitudes, whereas academic clinicians and veterinary nurses identified areas of concern. Although we did not assess the impact of targeted interventions, addressing these role‐based differences may strengthen safety culture and potentially improve patient outcomes. Future research should test these interventions, extend our findings to diverse geographical contexts and further develop strategies for fostering a robust culture of safety across the veterinary profession.

## Conflicts of interest and sources of funding

The authors declare no conflicts of interest or sources of funding for the work presented here.

## Data Availability

The data that support the findings of this study are available on request from the corresponding author. The data are not publicly available due to privacy or ethical restrictions.
